# Vitamin D and Bone Health of Older Adults within Care Homes: An Observational Study

**DOI:** 10.3390/nu14132680

**Published:** 2022-06-28

**Authors:** Orlagh Feehan, David J. Armstrong, Pamela J. Magee, Kirsty L. Pourshahidi, J. J. Strain, Laura Beggan, Diego F. Cobice, Emeir M. McSorley

**Affiliations:** 1 Nutrition Innovation Centre for Food and Health (NICHE), Ulster University, Coleraine BT52 1SA, UK; feehan-o@ulster.ac.uk (O.F.); david.armstrong@westerntrust.hscni.net (D.J.A.); pj.magee@ulster.ac.uk (P.J.M.); k.pourshahidi@ulster.ac.uk (K.L.P.); jj.strain@ulster.ac.uk (J.J.S.); beggan-l@ulster.ac.uk (L.B.); 2 Department of Rheumatology, Altnagelvin Hospital, Western Health and Social Care Trust, Londonderry BT47 6SB, UK; 3 Mass Spectrometry Centre, Biomedical Sciences Research Institute (BMSRI), Ulster University, Coleraine BT52 1SA, UK; d.cobice@ulster.ac.uk

**Keywords:** vitamin D deficiency, aged, 80 and over, nursing home, osteoporosis, vitamin D supplementation

## Abstract

Limited studies have reported vitamin D status and health outcomes in care home residents, a group at risk of vitamin D deficiency. This study investigated serum 25-hydroxyvitamin D (25-OHD) concentrations in older adults within care homes in Northern Ireland (NI) and its association with musculoskeletal health (ultrasound T-score, muscle strength, Timed Up & Go test (TUG)), bone turnover markers (BTMs), and immune function markers. A total of 87 participants were recruited with mean ± SD age 83.2 ± 7.9 years. Mean ± SD serum 25-OHD concentration (*n* 69) was 49.52 ± 35.58 nmol/L. Vitamin D deficiency (25-OHD <25 nmol/L) was observed in 34.8% (*n* 24) of participants with 17.4% (*n* 12) classified as insufficient (25-OHD 25–50 nmol/L) and 47.8% (*n* 33) as sufficient (25-OHD >50 nmol/L). 25-OHD concentration was not an independent predictor of T-score, muscle strength, TUG, or inflammatory cytokines. After adjusting for covariates, a significant negative association was observed between 25-OHD concentration and the BTMs; osteocalcin (β = −0.395; *p* = 0.001), procollagen type 1 N propeptide (P1NP) (β = −0.320; *p* = 0.012), and C-terminal telopeptide of type 1 collagen (CTX) (β = −0.377; *p* = 0.003). Higher 25-OHD concentration was positively associated with use of vitamin D ± calcium supplementation (β = 0.610; *p* < 0.001). Vitamin D deficiency and insufficiency were highly prevalent in this sample of care home residents in NI. Higher 25-OHD concentration was associated with greater supplement use and with reduced bone turnover, which in this population is linked with reduced bone loss. These findings emphasize the need for a mandatory vitamin D ± calcium supplementation policy specific for care home residents.

## 1. Introduction

Older adults, in particular those residing in care homes, are vulnerable to vitamin D deficiency [1,2,3], yet few studies have reported on the vitamin D status or its relationship to health outcomes in this vulnerable group. Limited evidence to date in care home residents from across the world suggests a high prevalence of vitamin D deficiency (25-hydroxyvitamin D (25-OHD) <50 nmol/L) [1,2,3,4,5]. Other observational studies have reported a mean 25-OHD concentration well below the 25 nmol/L cut-off [6,7,8]. In New Zealand, high 25-OHD concentrations (mean 25-OHD 89.9 nmol/L) have been reported in a small sample of aged-care residents, albeit this finding was attributed to a universal government vitamin D supplementation program where 75% of residents had received 50,000 international units (IU) vitamin D_3_/month [9]. Care home residents in northern latitudes such as Northern Ireland (NI) are at a greater risk of deficiency owing to the lack of dermal synthesis of vitamin D during the winter months [10]. There is no evidence to date reporting on the 25-OHD concentration of care home residents in NI, with only one study in the Republic of Ireland identifying that some 42% of care home residents were vitamin D deficient (25-OHD <25 nmol/L) [11]. It is important to note that the retrospective observational nature of this study within the Republic of Ireland was a significant limitation; with no information available on past medical history, medication use or supplement use, as stated in the discussion of the paper.

A low vitamin D status results in decreased calcium absorption, elevated parathyroid hormone, and increased rates of bone resorption [12]. Vitamin D can be obtained from limited sources within the diet, and the main source is exposure to UV sunlight. With advancing age the ability to synthesize vitamin D from sunlight exposure becomes compromised [13] which in combination with additional factors, including poor dietary intake of vitamin D, impaired absorption and hydroxylation, compromised renal function, and medication use [14], as well as reduced time spent outdoors, can lead to low circulating concentrations of 25-OHD and thus vitamin D deficiency in care home residents. It is proposed that a significant number of care home residents have undiagnosed and undertreated osteoporosis, and that this may be partly attributed to undetected vitamin D deficiency [15]. An optimal 25-OHD concentration is also important to support the immune system [16,17] and has been associated with lower markers of inflammation [18] as well as quicker recovery from infections [19]. Furthermore, the immunomodulatory activity of vitamin D is suggested to enhance bone density by favorably altering the microbiota composition to reduce bone mass loss [20,21].

Studies to date have not always reported or considered additional factors which impact 25-OHD concentration and the exclusion of individuals with cognitive impairment/dementia is likely to bias the outcomes owing to the cohort not being truly representative of the population group [22,23,24]. Therefore, the aim of this study was to investigate the 25-OHD concentration of older adults within care homes in NI and to determine its relationship with musculoskeletal health and immune function markers. Secondary analysis investigated factors that impact 25-OHD concentration. We hypothesize that there will be a high prevalence of vitamin D insufficiency/deficiency within care home residents and that low 25-OHD concentrations will be associated with impaired musculoskeletal health and immune function.

## 2. Materials and Methods

### 2.1. Study Participants

**Figure 1:**
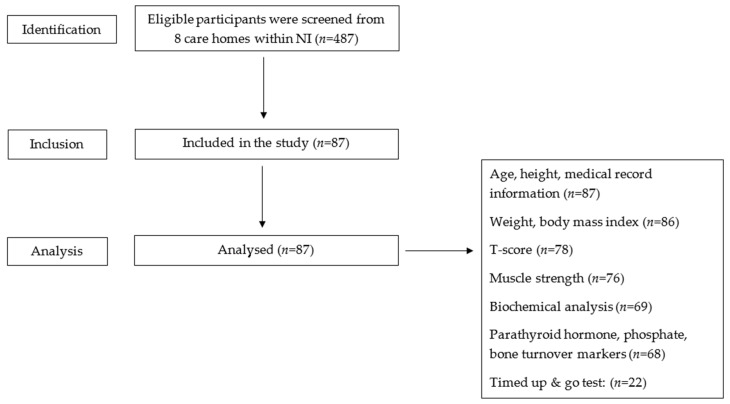
Strobe flow diagram of observation study.

From May 2019 to March 2020, care homes from across the Western Health and Social Care Trust (WHSCT) in NI were contacted and 8 care homes agreed to partake in the study (Figure 1). Older adults (≥65 years) within these 8 care homes were approached to participant in the study. An estimate of the sample size for this cross-sectional study was based on a rate of 40% vitamin D deficiency (25-OHD <25 nmol/L) as reported by the Scientific Advisory Committee for Nutrition (SACN) in their vitamin D and health report [10]. With an expected frequency of 40% for the percentage deficient and an acceptable margin of error of ±7% in the 95% confidence interval, a sample size of 180 participants was calculated; however, recruitment ceased in March 2020 owing to the global SARS-CoV-2 (COVID-19) pandemic, and recruitment was curtailed at 87 participants, with 25-OHD concentrations available for 69 participants. Results from this study identified 24 out of 69 participants with vitamin D deficiency giving an estimate of 35% (95% CI 24% to 47%). The sample was well-characterized, with participants recruited from several care homes from different geographical locations within the WHSCT and included residents with cognitive impairment/dementia.

Inclusion criteria, determined using a screening questionnaire, included residing in the care home for >1 month, ≥65 years old, were not end of life and had the ability to provide informed assent and written consent. In the case of an Adult Lacking in Capacity (ALC), the multi-disciplinary team at the care home determined their involvement in the study if it was deemed beneficial to their care.

Ethical approval was obtained from the Office for Research Ethics Committees Northern Ireland (ORECNI) and was conducted in accordance with the Declaration of Helsinki (IRAS ID: 227739, REC reference number: 18/NI/0114, ClinicalTrials.gov Identifier: NCT04399291). 

### 2.2. Physical Examination

Most recent measures of height (m) and weight (kg) were collected from care home care records to determine Body Mass Index (BMI, kg/m^2^). In the case where height was not available, ulna arm length measurement was taken, and height calculated using conversion tables [25]. BMI categories were defined as underweight (<18 kg/m^2^), normal (18.5 kg–24.9 kg/m^2^), overweight (25–29.9 kg/m^2^), and obese (>30 kg/m^2^). As an estimate of muscle strength, grip strength (kg) was measured on the non-dominant hand using a hand-grip dynamometer (Stoelting, Illinois (IL), United States of America (USA)). A second measurement was taken on the same arm, and an average of both readings calculated. T-score (SD) was measured using a portable Achilles^TM^ quantitative ultrasound (QUS) system scan of the heel (os calcis). Bone ultrasonometers have been shown to be a quick and affordable method of initial assessment of osteoporosis and fracture risk which is particularly useful in this cohort where Dual-energy X-ray absorptiometry (DXA) scanning is not a practical option. A Timed Up & Go (TUG) test was conducted to test basic mobility in those who were physically able to complete the test [26].

### 2.3. Health and Lifestyle Questionnaire

Participants provided information on lifestyle and general health including history of fracture, alcohol intake, smoking status, and level of mobility. Information on past medical history including a diagnosis of osteoporosis recorded in medical records, dementia/cognitive impairment, vitamin D ± calcium supplement use, vitamin D supplementation dose, fracture history, and use of medication known to affect bone health were collected from hospital records by the study clinical lead. 

### 2.4. Biochemical Data Collection

#### 2.4.1. General Clinical Chemistry

A full blood count was completed using a Sysmex automated analyzer (Sysmex, Milton Keynes, United Kingdom (UK)) at Ulster University. Liver, bone, and electrolyte profiles were completed on non-fasting serum samples using a Roche Modular Analyzer. Parathyroid hormone (PTH) was measured in plasma using a fully automated clinical chemistry and immunoassay system. Liver, bone, electrolyte, and PTH analysis was conducted by Altnagelvin Hospital Clinical Biochemistry Laboratory.

#### 2.4.2. Plasma 25-OHD

Vitamin D metabolites 25-hydroxyvitamin D2 (25-OHD2) and 25-hydroxyvitamin D3 (25-OHD3) (ng/mL) were analyzed in non-fasting plasma samples using an ICH validated pre-column derivatization with 4-Phenyl-1,2,4-triazoline-3,5-dione (PTAD) coupled with high-performance liquid chromatography–tandem mass spectrometry (HPLC-MS/MS) method at Ulster University. This method is currently regarded as the ‘gold-standard’ technique for analysis of vitamin D metabolites. Chromatographic separation was performed using a Shimadzu Nexera UHPLC system (Kyoto, Japan) using a polar C18 column (Phenomenex, Torrance, CA, United States of America (USA)) in a gradient mode from 50% acetonitrile with 0.1% *v/v* formic acid to 100% acetonitrile achieving a run time of 15 min. Injection volume was set to 20 µL and column temperature at 40 °C. Mass spectrometry detection was accomplished by an API 4000 triple quadrupole mass spectrometer (SCIEX, Macclesfield, UK) using a turbo ion spray in positive ion mode. Each metabolite was detected and quantified based on a previously optimized multiple reaction monitoring transition (MRM). The MRM transition for 25-OHD3 was *m*/*z* 558.3→*m*/*z* 298.2 and for 25-OHD2 was *m*/*z* 570.3→*m*/*z* 298.2. For the internal standard (25(OH) D3-d6) MRM transition *m*/*z* 564.4→*m*/*z* 298.2 was used. Duplicate injections were made of each sample preparation and analysis reported the average (ng/mL) of two injections. Dynamic range for 25-OHD3 and (25-OHD2) was from 2.5 ng/mL to 200 ng/mL and the Lower Limit of Quantification (LLOQ) of the method was 5 ng/mL for 25-OHD2 and 2.5 ng/mL for 25-OHD3. The quality and accuracy of the method was monitored using the National Institute of Standards and Technology (NIST) 972 vitamin D standard reference material and pooled samples. All study CV% were below 15 as per method criteria. CVs were between 5% and 8% for 25-OHD3 and 8–12% for 25-OHD2 positives (above LLOQ). QCs were 6–9% with back calculated accuracy of 92–105% for 25-OHD3. QCs were 7–10% with back calculated accuracy of 95–103% for 250 HD2. Vitamin D results were converted from ng/mL to nmol/L by multiplying ng/mL by 2.5. Vitamin D deficiency was classified as 25-OHD concentrations <25 nmol/L, insufficiency 25-OHD concentrations between 25 and 50 nmol/L and sufficiency 25-OHD concentrations >50 nmol/L.

#### 2.4.3. Bone Turnover Marker Analysis

Bone turnover markers (BTMs) total osteocalcin (OC; turnover marker), C-terminal telopeptide of type 1 collagen (CTX; resorption marker), and procollagen type 1 N propeptide (P1NP; formation marker) were measured in non-fasting serum samples using Roche kits at St James’s Hospital, Dublin, Ireland. For total OC, within run precision was 0.8% at 19.9 ng/mL and between run precision 5.0% at 18.8 ng/mL and 6.7% at 95.3 ng/mL. The reference range is 14–46 ng/mL for males 50–70 years and 15–46 ng/mL for postmenopausal women (no hormone replacement therapy) and 13–48 ng/mL for osteoporotic women. For CTX, the within run precision was 1.9% at 0.30 ng/mL and between run precision 1.9% at 0.35 ng/mL and 2.4% at 0.78 ng/mL. The reference range is <1.008 ng/mL for postmenopausal women and <0.704 for males 50–70 years, and <0.854 ng/mL males >70 years. For P1NP, the within run precision was 1.2% at 31.5 ng/mL and the between run precision was 4.1% at 27.4 ng/mL and 4.3% at 166 ng/mL. The reference range is 15–90 ng/mL for postmenopausal women and 15–80 ng/mL for males 25–70 years and 15–115 ng/mL for males >70 years.

### 2.5. Immune Function Analysis

The immune markers (C-reactive protein (CRP), interferon gamma (IFN-γ), tumour necrosis factor alpha (TNF-α) and interleukins (IL): IL-1 β, IL-2, IL-4, IL-6, IL-8, IL-10, IL-12p70, and IL-13 were analyzed in non-fasting serum samples using Meso Scale Discovery (MSD) multiplex assay (CVs: 3.96%, 5.93%, 6.71%, 16.24%, 7.63%, 4.01%, 2.89%, 2.51%, 6.96%, 8.01%, 13.79%, respectively). The immune markers included cytokines strongly associated with the innate immune response to infection (CRP, IL-1 β, IL-6, IL-8, TNF-α), those with anti-inflammatory functions (IL-4, IL-10, IL-13), immune tolerance and regulation (IL-2, IL-12p70), and those involved in anti-viral responses (IFN-γ). All markers of inflammation were measured as pg/mL, except for CRP which was measured in mg/L. For undetectable cytokines falling below the lower limit of detection, LLOD/√2 values were inputted as a replacement value [27].

### 2.6. Statistical Methods

All data analyses were performed using Statistical Package for the Social Sciences (SPSS, IBM SPSS Statistics version 26). Nominal data were presented as mean (standard deviation (SD)) and categorical variables presented as frequencies (*n*) and percentage (%). For the purpose of statistical analysis, participants who were prescribed just calcium (*n* 3) and just vitamin D supplementation (*n* 1) were combined with the vitamin D ± calcium supplement users group. Data was tested for normality using Kolmogorov–Smirnov statistic test.

The primary outcome of this study was to determine 25-OHD concentration and the prevalence of vitamin D deficiency (25-OHD < 25 nmol/L), insufficiency (25-OHD 25–50 nmol/L), and sufficiency (25-OHD > 50 nmol/L) for the cohort. Mann–Whitney U analysis was used to compare characteristics between sex, 25-OHD concentration <50 and >50 nmol/L, those prescribed vitamin D ± calcium supplement, and those not prescribed supplementation. Secondary analyses using multiple regression analysis were performed to investigate associations between 25-OHD concentration and musculoskeletal health parameters, BTMs, and cytokines. BMI, sex, and age were chosen as covariates for analysis due to their known association with 25-OHD concentration and musculoskeletal health parameters, BTMs, and inflammatory cytokines.

A multivariate general linear regression model was used to determine predictors of 25-OHD concentration, musculoskeletal health parameters, BTMs, and inflammatory cytokines. Predictors were entered simultaneously in two blocks. BMI, sex, age (model 1), and 25-OHD concentration (model 2) were entered into the model for musculoskeletal health parameters, BTMs, and inflammatory cytokines. BMI, sex, age (model 1), vitamin D ± calcium supplement use (model 2) were entered into the model for 25-OHD concentration. The Benjamini–Hochberg procedure (20% false discovery rate) was applied to reduce the potential for type 1 error. Sensitivity analysis was conducted to determine if there were any differences in using transformed data and untransformed data for regression analysis. It was found that using the untransformed data led to less skewed data and therefore untransformed data were presented. Significance was set at *p* < 0.05 throughout.

## 3. Results

### 3.1. Participant Characteristics

Biochemical analysis was available for 69 participants (*n* 68 for BTMs and CRP) due to refusal of blood sampling at appointment (*n* 12) or inability to obtain blood sample (*n* 6). When comparing characteristics between participants who did have a blood sample available and those who did not, there was a significant difference in mean ± SD age in which those who provided a blood sample were significantly older (84.2 ± 7.3 vs. 79.4 ± 9.1 years, *p* = 0.04, respectively). Table 1 and Table 2 summarize the characteristics of the cohort. Mean ± SD age and BMI for the total cohort were 83.21 ± 7.9 years and 27.87 ± 7.42 kg/m^2^, respectively. Weight, T-score, and muscle strength were significantly higher in males compared with females.

A total of 32.2% of the cohort had osteoporosis recorded in medical records, whereas QUS heel scan results identified osteoporosis in 64.1% of the cohort. Some 44.8% of participants had a history of low trauma fracture. Prescribed medications known to affect bone health included proton pump inhibitors (43.7%), bisphosphonates (19.5%), statins (55.2%), beta-blockers (21.8%), and anti-epileptics (13.8%). Out of the 87 participants, 49.4% (*n* 43) were prescribed vitamin D ± calcium supplements of which 42.5% (*n* 37) were prescribed a supplement dose of 800 IU vitamin D_3_/day. A total of 34.5% of participants were chairbound, 32.2% were mobile to some extent, and 33.3% were mobile to some extent outdoors. The majority of participants (91.3%) spent <30 min outside each day. Median (IQR) CRP was 8.85 (179.8) mg/L and 34 participants with a CRP >10 mg/L. As expected, CRP was strongly correlated with IL-6 (r = 0.635; *p* < 0.001) reflecting innate immune responses and inflammation.

### 3.2. OHD Concentration

**Figure 2:**
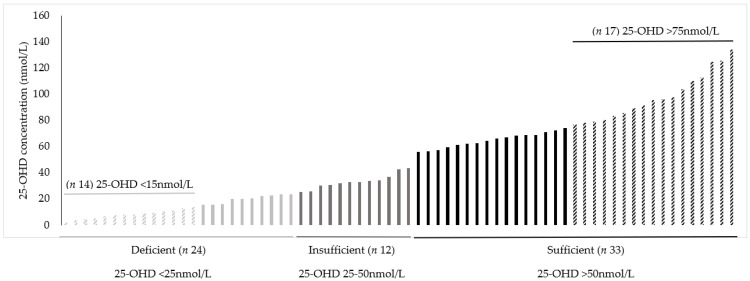
Plasma 25-OHD concentration (nmol/L) of (*n* 69) participants within deficient, insufficient and sufficient vitamin D status categories.

Mean ± SD 25-OHD concentration for the total group was 49.52 ± 35.58 nmol/L with a median ± interquartile range of 42.75 ± 57.58 nmol/L. Within the total group, 34.8% were classified as deficient (25-OHD <25 nmol/L), 17.4% were insufficient (25–50 nmol/L), and 47.8% were sufficient (25-OHD >50 nmol/L). Some 20.3% of participants had 25-OHD concentration <15 nmol/L and 24.6% had 25-OHD concentration >75 nmol/L (Figure 2). There was no significant difference in 25-OHD concentration between males and females (45.21 ± 35.03 vs. 52.29 ± 36.07 nmol/L). Those with a 25-OHD concentration >50 nmol/L had a significantly lower PTH and total alkaline phosphatase concentration compared to those with 25-OHD concentration <50 nmol/L.

### 3.3. Predictors of Physical and Biochemical Parameters

Current 25-OHD concentration was not a significant predictor of T-score, muscle strength, TUG or any of the measured inflammatory cytokines with and without adjusting for covariates (Table 3). A higher 25-OHD concentration was associated with lower bone turnover as demonstrated by significant negative associations with osteocalcin (β = −0.395; *p* = 0.001), P1NP (β = −0.320; *p* = 0.012), and CTX (β = −0.377; *p* = 0.003) in both the model adjusted for covariates and without adjustment. The results remained significant after applying the Benjamini–Hochberg procedure. Being female was a significant negative predictor of T-score (β = −0.439; *p* = <0.001) and muscle strength (β = −0.582, *p* = <0.001) and a positive predictor of a higher IL-12p70 concentration (β = 0.316; *p* = 0.01) (Table 4). A higher BMI was associated with a significantly higher TUG score (β = 0.497, *p* = 0.030) after adjustment for covariates and a higher IL-10 concentration with and without adjustment for covariates (β = 0.268, *p* = 0.030). 

### 3.4. Predictors of 25-OHD Concentration

BMI, sex and age were not significant predictors of 25-OHD concentration in either model; however, being prescribed a vitamin D ± calcium supplement was significantly associated with higher 25-OHD concentration (β = 0.610, *p* = <0.001) and together accounted for 40% of the variance in 25-OHD concentration (Table 3).

**Figure 3:**
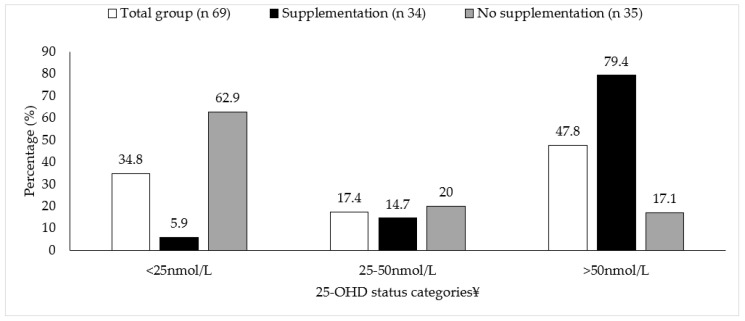
Vitamin D ± calcium supplement use by category of plasma 25-OHD concentration. ^¥^ Categories for 25-OHD concentration: deficient (<25 nmol/L), insufficient (25-50 nmol/L), sufficient (>50 nmol/L).

Overall, in the entire cohort, those prescribed vitamin D ± calcium supplements had a significantly greater 25-OHD concentration than those who were not prescribed supplements (71.74 ± 27.23 vs. 27.93 ± 28.96 nmol/L, *p* < 0.001, respectively). Vitamin D ± calcium supplement use by category of 25-OHD concentration is shown in Figure 3. Vitamin D ± calcium supplement users had a significantly lower alkaline phosphatase, PTH, osteocalcin, P1NP and CTX compared to non-supplement users (*p* < 0.05) (data not shown). 

## 4. Discussion

This study provides the first evidence for a high prevalence of vitamin D deficiency and insufficiency in care home residents in NI. Of concern, we identified a significant number of residents with extremely low 25-OHD (20.3% 25-OHD <15 nmol/L) which is known to have negative implications for musculoskeletal health and immune responses. In this cohort, some 47.8% of participants achieved 25-OHD concentration >50 nmol/L, with only 24.6% having a 25-OHD concentration above 75 nmol/L. Higher 25-OHD was attributed to the prescription of vitamin D ± calcium supplementation. Although 25-OHD concentration was not a predictor of T-score, muscle strength, TUG or any of the inflammatory cytokines, study recruitment was curtailed by the onset of the COVID-19 pandemic and therefore the sample size may not have been sufficient to test for these associations. It is worth noting that participants identified with osteoporosis and appropriately treated would usually be prescribed vitamin D, and these subjects would therefore have high 25-OHD concentrations and a low T-score due to osteoporosis. Conversely, participants with undiagnosed osteoporosis, would have a low T-score and most likely a low 25-OHD concentration. This relationship may explain why no association between 25-OHD concentration and T-score was observed in the current study. We did observe significant associations between higher 25-OHD and lower bone turnover and PTH supporting a role for vitamin D in maintaining bone health in this vulnerable group.

The mean 25-OHD concentration was 49.52 ± 35.58 nmol/L which is similar to that reported by other studies of care home residents from various geographical regions [28,29,30]. Mean 25-OHD concentration was substantially higher in supplement users compared to non-supplement users (71.74 vs. 27.93 nmol/L, respectively); nevertheless, some 52.9% of residents were not receiving any form of vitamin D supplementation irrespective of the 10 µg/day recommended by SACN in the UK, which is cause for concern. In addition, observation studies of care home residents show that dietary intake of vitamin D is as low as 1.27 µg/day and sunlight exposure as little as a couple of minutes per day [1] placing them at high risk of year-round vitamin D deficiency. Within our study, sunlight exposure was limited, with 91.3% self-reporting spending <30 min outside per day and collectively highlights the need for measures to enhance vitamin D supplementation within this at-risk group.

Vitamin D deficiency up to 80% [5] and even over 90% [1,4] has been reported in other cohorts of care home residents; however, these studies used a cut-off of 25-OHD <50 nmol/L to define deficiency. Based on a cut-off of <50 nmol/L, prevalence of vitamin D deficiency would increase from 34.8% to 52.2% in our study. It is evident from the available literature and our own data, that a more meaningful picture can be drawn from describing the rates of vitamin D deficiency rather than mean 25-OHD concentration when considering care home residents. Mean 25-OHD concentration, which in this study is close to sufficiency, may conceal the fact that many residents are vitamin D deficient and prevent the introduction of vitamin D supplementation for all residents to ensure 25-OHD concentrations reach sufficiency.

Our findings show that a greater 25-OHD concentration was a significant negative predictor of BTMs; osteocalcin, P1NP and CTX, which has also been observed in care home residents in Spain and Argentina [1]. In addition to our finding on BTMs, PTH concentration was twice as high in participants with a 25-OHD concentration <50 nmol/L compared to those with a 25-OHD concentration >50 nmol/L. Given the established role of PTH in calcium homeostasis and its action to stimulate bone remodeling, these findings highlight the importance of maintaining a higher 25-OHD concentration for the prevention of bone loss in care home residents.

Similar to our results, others have found 25-OHD concentration is significantly higher, and rates of deficiency are lower in cohorts where vitamin D supplement use is greater [9,30]. The use of vitamin D supplements is frequently not reported in care home studies [2,11,31,32] or often participants are excluded from observational studies if they are on supplementation [1,5,29,33] making it difficult to interpret the true prevalence of vitamin D deficiency in care home residents. Vitamin D supplement use has been shown to be as low as <10% in some cohorts of care home residents [34,35,36]. Vitamin D ± calcium supplementation was shown to be a significant predictor of a higher 25-OHD concentration. From the 41 participants prescribed vitamin D, 37 were prescribed 800IU (20 µg dose) which suggest the SACN recommendation of 10µg per day may not be sufficient to adequately increase status in this age group.

Observational studies of care home residents have reported varying rates of osteoporosis within the literature (19.7%, 25.4%, 47.3%) [31,34,37]. Our study showed that following heel scan with the bone ultrasonometer, twice as many residents had osteoporosis compared to known osteoporosis from their medical records. Our findings show that four residents with known osteoporosis from medical records were not receiving vitamin D ± calcium supplementation compared to 21 residents that had osteoporosis based on QUS heel scan who were not receiving supplementation. These findings suggest that osteoporosis may be underdiagnosed and undertreated in this group and using a portable bone ultrasonometer may play a role in diagnosing osteoporosis in residents unable to attend for a full DXA scan.

This study has presented data in a well-characterized cohort of care home residents, with information collected on several robust outcome parameters that were comparable with other observational studies conducted in this research area [7,31,37,38,39]. Moreover, this study is the first to report on the 25-OHD concentration of care home residents in NI. This study included participants who had cognitive impairment/dementia, a vulnerable group often excluded from observational studies of care home residents. In addition, our study included participants who were taking vitamin D ± calcium supplementation and are often frequently excluded from research studies. Recruitment and sample size were limited by the COVID-19 pandemic; nevertheless, this paper provides added important clinical data which enhances the limited body of evidence available on predictors of physical and biochemical parameters of health in care home residents. Future research using a larger cohort designed to assess outcome measures at multiple time points is required to validate our findings. Owing to the nature of the study all participants provided non-fasting blood samples which should be considered when interpreting the findings.

Within this cohort of care home residents in NI, there was a high prevalence of vitamin D deficiency and insufficiency and a greater 25-OHD concentration was associated with reduced bone turnover which is important for the prevention of bone loss and maintenance of bone health in this group. Many residents without a formal diagnosis of osteoporosis had a low T-score as measured by QUS heel scan. Mean 25-OHD concentration is not the most appropriate way to describe vitamin D status in a population, owing to the significant variation in 25-OHD concentration between those prescribed vitamin D ± calcium supplementation and those who were not. Prescription of vitamin D ± calcium supplements was strongly associated with a higher 25-OHD concentration, albeit many residents do not receive calcium or vitamin D supplementation. These findings strongly support the arguments for mandatory supplementation policy specific for care home residents or introduction of fortified foods to prevent vitamin D deficiency.

## Figures and Tables

**Table 1:** Supplement use and musculoskeletal health of care home residents.

Descriptive	*n* Frequency (%)
**Vitamin D/calcium use**	***n* 87**
Vitamin D + calcium	40 (46.0)
No supplementation	43 (49.4)
Calcium alone	3 (3.4)
Vitamin D alone	1 (1.2)
**Vitamin D dose**	
No Supplementation	46 (52.9)
200 IU	1 (1.15)
400 IU	2 (2.3)
500 IU	1 (1.15)
800 IU	37 (42.5)
**T Score Category ^¥^**	***n* 78**
Normal	11 (14.1)
Osteopenia	17 (21.8)
Osteoporosis	50 (64.1)
**Known Osteoporosis ^¤^**	
Yes	28 (32.2)
No	59 (67.8)
**History of Low Trauma Fracture**	***n* 87**
Yes	39 (44.8)
No	48 (55.2)
**No of Low Trauma Fractures**	
1	25 (28.7)
2	7 (8.1)
3	4 (4.6)
>3	3 (3.4)
No history of fracture	48 (55.2)
**Dementia/Cognitive Impairment**	
Yes	30 (34.5)
No	57 (65.5)
**BMI Category**	***n* 86**
<18.5 kg/m^2^	2 (2.3)
18.5–24.9 kg/m^2^	38 (44.2)
25–29.9 kg/m^2^	17 (19.8)
>30 kg/m^2^	29 (33.7)

Data Presented as Frequency (Percentage). IU; international units, BMI; Body Mass Index. **^¥^** T-score based on heel ultrasound assessment. **^¤^** Recorded by medical practitioner from medical records.

**Table 2:** Descriptive characteristics of care home residents.

Descriptives	Total Cohort	Males	Females	25-OHD <50 nmol/L	25-OHD >50 nmol/L
	***n* 87**	***n* 35**	***n* 52**	***n* 36**	***n* 33**
Age (years)	83.21 ± 7.9	81.29 ± 8.57	84.5 ± 7.2	83.89 ± 7.13	84.52 ± 7.56
Height (m)	1.65 ± 0.11	1.72 ± 0.09	1.6 ± 0.08 **	1.64 ± 0.10	1.65 ± 0.11
	***n* 86**	***n* 34**	***n* 52**	***n* 35**	***n* 33**
Weight (kg)	75.94 ± 21.4	81.45 ± 20.66	72.33 ± 21.3 *	78.81 ± 19.81	73.56 ± 21.95
Body Mass Index (kg/m^2^)	27.87 ± 7.42	27.46 ± 6.32	28.3 ± 8.1	29.51 ± 7.6	26.88 ± 6.6
	***n* 78**	***n* 30**	***n* 48**	***n* 32**	***n* 31**
T Score ^¥^	−2.8 ± 1.61	−1.89 ± 1.68	−3.4 ± 1.25 **	−2.7 ± 1.53	−2.76 ± 1.83
	***n* 76**	***n* 31**	***n* 45**	***n* 32**	***n* 30**
Muscle strength (kg)	12.37 ± 5.88	16.49 ± 6.38	9.52 ± 3.3 **	13.6 ± 5.99	11.92 ± 6.43
	***n* 22**	***n* 11**	***n* 11**	***n* 9**	***n* 11**
TUG (seconds)	39.17 ± 14.24	37.32 ± 16.04	41.03 ± 12.7	39.02 ± 12.85	39.15 ± 15.13
**Biochemical analysis**	***n* 69**	***n* 27**	***n* 42**	***n* 36**	***n* 33**
25-OHD (nmol/L)	49.52 ± 35.58	45.21 ± 35.03	52.29 ± 36.07	19.8 ± 11.7	81.94 ± 21.5 **
Alkaline Phosphatase (U/L)	103.42 ± 58.4	98.85 ± 29.11	106.36 ± 71.39	115.69 ± 73.36	90.03 ± 31.73 *
AST (U/L)	19.35 ± 10.39	19.3 ± 9.28	19.38 ± 11.15	17.92 ± 9.9	20.91 ± 10.82
ALT (U/L)	15.09 ± 9.39	17.67 ± 9.34	13.43 ± 9.15 *	15.69 ± 11.46	14.42 ± 6.54
GGT (U/L)	47.23 ± 57.81	37.78 ± 32.33	53.31 ± 69.16	54.36 ± 53.34	49.27 ± 63.12
Urea (mmol/L)	8.4 ± 3.33	8.08 ± 2.97	8.6 ± 3.56	8.39 ± 2.92	8.39 ± 3.77
Creatinine (mmol/L)	99.28 ± 37.7	104.07 ± 31.91	96.19 ± 41.06	97.31 ± 34.72	101.42 ± 41.13
Est GFR (mL/min)	51 ± 11.18	53.26 ± 9.10	49.55 ± 12.22	51.44 ± 11.28	50.52 ± 11.23
Adjusted Calcium (mmol/L)	2.26 ± 0.15	2.24 ± 0.10	2.27 ± 0.17 *	2.26 ± 0.08	2.26 ± 0.20
	***n* 68**	***n* 27**	***n* 41**	***n* 36**	***n* 32**
PTH (ng/L)	76.62 ± 56.86	65.05 ± 45.62	84.24 ± 62.56	101.47 ± 65.48	48.66 ± 24.96 **
Phosphate (mmol/L)	1.16 ± 0.17	1.13 ± 0.21	1.17 ± 0.13	1.15 ± 0.20	1.16 ± 0.12
**Markers of inflammation**	***n* 69**	***n* 27**	***n* 42**	***n* 36**	***n* 33**
TNF-α (pg/mL)	4.87 ± 2.03	4.69 ± 1.99	4.98 ± 2.07	4.84 ± 1.82	4.89 ± 2.26
IFN-γ (pg/mL)	31.95 ± 106.86	12.01 ± 15.16	44.78 ± 135.51	29.72 ± 85.81	34.39 ± 127.28
IL-1B (pg/mL)	0.21 ± 0.22	0.24 ± 0.24	0.19 ± 0.21	0.22 ± 0.22	0.19 ± 0.23
IL-2 (pg/mL)	0.73 ± 1.63	0.49 ± 0.55	0.89 ± 2.04	0.7 ± 1.78	0.77 ± 1.48
IL-4 (pg/mL)	0.02 ± 0.02	0.02 ± 0.02	0.02 ± 0.01	0.02 ± 0.01	0.02 ± 0.02
IL-6 (pg/mL)	3.36 ± 3.77	3.95 ± 5.17	2.98 ± 2.49	3.06 ± 2.98	3.69 ± 4.5
IL-8 (pg/mL)	36.27 ± 33.89	29.56 ± 29.36	40.58 ± 36.2	38.87 ± 36.69	33.43 ± 30.86
IL-10 (pg/mL)	0.88 ± 2.58	1.18 ± 3.89	0.69 ± 1.17	1.21 ± 3.51	0.53 ± 0.62
IL-12p70 (pg/mL)	0.21 ± 0.24	0.12 ± 0.07	0.27 ± 0.29 *	0.21 ± 0.2	0.20 ± 0.27
IL-13 (pg/mL)	0.51 ± 0.35	0.5 ± 0.42	0.52 ± 0.30	0.54 ± 0.39	0.48 ± 0.31
	***n* 68**	***n* 27**	***n* 41**	***n* 35**	***n* 33**
CRP (mg/L)	24.85 ± 37.66	26.87 ± 43.44	23.51 ± 33.84	22.98 ± 30.8	26.83 ± 44.21
**Bone Turnover Markers**	***n* 68**	***n* 26**	***n* 42**	***n* 35**	***n* 32**
Osteocalcin (ng/mL)	28.16 ± 20.18	24.05 ± 14.17	35.7 ± 22.93	35.05 ± 23.52	19.48 ± 9.98 **
P1NP (ng/mL)	62.63 ± 45.63	56.75 ± 29.32	66.27 ± 53.32	75.76 ± 54.42	47.17 ± 27.74 *
CTX (ng/mL)	0.45 ± 0.27	0.41 ± 0.22	0.47 ± 0.30	0.53 ± 0.29	0.34 ± 0.18 **

Data Presented Mean ± Standard deviation. **^¥^** T-score based on heel ultrasound assessment. TUG; Timed up and Go Test, 25-OHD; 25-hydroxyvitamin D, Est GFR; estimated glomerular filtration rate, AST; aspartate aminotransferase, ALT; alanine transaminase, GGT; gamma-glutamyl transferase, PTH; parathyroid hormone, TNF-α; tumour necrosis factor alpha, IFN-γ; interferon gamma, IL-1B; interleukin-1-beta, IL-2, 4, 6, 8, 10, 12, 13; interleukin 2, 4, 6, 8, 10, 12, 13, P1NP; total procollagen type 1 N-terminal propeptide, CTX; C-terminal telopeptide of type 1 collagen, CRP; C-reactive protein. *p* value obtained from Mann–Whitney U test. *p* < 0.05 * and *p* < 0.001 ** denotes significance.

**Table 3:** Multiple regression showing associations between 25-OHD concentration and physical and biochemical parameters in care home residents.

Dependant	Model ^1^	Predictor Variables	R^2^	Standardized β	*p* Value
T-score	1	BMI		−0.102	0.332
		Sex		−0.439	<0.001 *
		Age	0.246	−0.152	0.150
	2	25-OHD	0.299	0.023	0.840
Muscle strength (kg)	1	BMI		0.190	0.054
		Sex		−0.582	<0.001 *
		Age	0.380	−0.061	0.539
	2	25-OHD	0.475	−0.073	0.475
TUG (seconds)	1	BMI		0.497	0.030 *
		Sex		0.058	0.778
		Age	0.270	0.283	0.190
	2	25-OHD	0.277	0.066	0.785
Osteocalcin (ng/mL)	1	BMI		0.090	0.477
		Sex		0.150	0.238
		Age	0.038	0.082	0.523
	2	25-OHD	0.202	−0.395	0.001 *
P1NP (ng/mL)	1	BMI		0.127	0.317
		Sex		0.103	0.420
		Age	0.026	−0.021	0.870
	2	25-OHD	0.123	−0.320	0.012 *
CTX (ng/mL)	1	BMI		0.022	0.865
		Sex		0.106	0.408
		Age	0.015	0.045	0.730
	2	25-OHD	0.171	−0.377	0.003 *
25-OHD (nmol/L) ^¥^	1	BMI		−0.228	0.067
		Sex		0.083	0.508
		Age	0.058	−0.044	0.730
	2	Vitamin D ± calcium supplement use	0.413	0.610	<0.001 *

^1^ Model 1: Adjusting for BMI, sex and age; ^1^ Model 2: Further adjusting for 25-OHD concentration; ^1^ Model 2: Further adjusting for vitamin D ± calcium supplement use for 25-OHD concentration ^¥^ BMI; body mass index, β; beta-coefficient, TUG; timed up and go test, P1NP; procollagen type 1 N propeptide, CTX; C-terminal telopeptide of type 1 collagen, 25-OHD, plasma 25-hydroxyvitamin D. *p* < 0.05 * denotes significance.

**Table 4:** Multiple regression showing associations between 25-OHD concentration and inflammatory cytokines.

Dependant	Model ^1^	Predictor Variables	R^2^	Standardized β	*p* Value
TNF-α (pg/mL)	1	BMI		0.065	0.597
		Sex		0.072	0.568
		Age	0.045	0.179	0.163
	2	25-OHD	0.045	0.009	0.941
IFN-γ (pg/mL)	1	BMI		0.078	0.532
		Sex		0.138	0.281
		Age	0.029	0.050	0.695
	2	25-OHD	0.033	−0.058	0.652
IL-β (pg/mL)	1	BMI		0.109	0.383
		Sex		−0.088	0.491
		Age	0.020	0.010	0.938
	2	25-OHD	0.028	−0.094	0.465
IL-2 (pg/mL)	1	BMI		−0.036	0.772
		Sex		0.105	0.412
		Age	0.020	0.059	0.649
	2	25-OHD	0.026	−0.085	0.508
IL-4 (pg/mL)	1	BMI		−0.215	0.085
		Sex		−0.041	0.745
		Age	0.050	−0.068	0.595
	2	25-OHD	0.062	0.114	0.369
IL-6 (pg/mL)	1	BMI		0.108	0.388
		Sex		−0.138	0.281
		Age	0.031	0.082	0.521
	2	25-OHD	0.040	0.102	0.425
IL-8 (pg/mL)	1	BMI		0.016	0.897
		Sex		0.148	0.247
		Age	0.032	0.069	0.589
	2	25-OHD	0.065	−0.189	0.136
IL-10 (pg/mL)	1	BMI		0.268	0.030 *
		Sex		−0.087	0.485
		Age	0.08	0.011	0.930
	2	25-OHD	0.096	−0.133	0.285
IL-12p70 (pg/mL)	1	BMI		0.011	0.924
		Sex		0.316	0.012 *
		Age	0.099	−0.004	0.976
	2	25-OHD	0.103	−0.060	0.629
IL-13 (pg/mL)	1	BMI		−0.032	0.798
		Sex		0.051	0.692
		Age	0.004	−0.021	0.873
	2	25-OHD	0.009	−0.076	0.559
CRP (mg/L)	1	BMI		0.055	0.663
		Sex		−0.032	0.806
		Age	0.007	−0.043	0.741
	2	25-OHD	0.012	0.067	0.605

^1^ Model 1: Adjusting for BMI, sex and age; ^1^ Model 2: Further adjusting for 25-OHD concentration; BMI; body mass index, β; beta-coefficient, TNF-α; Tumor Necrosis Factor Alpha, IFN-γ; Interferon Gamma, IL-1B; Interleukin-1-Beta, IL-2, 4, 6, 8, 10, 12, 13; Interleukin 2, 4, 6, 8, 10, 12, 13. Vitamin D ± calcium supplement use vs. non-supplement use. *p* < 0.05 * denotes significance.

## Data Availability

Additional data are available from the corresponding author on reasonable request.
